# The ratio of QRS/RV_6_-V_1_: a new electrocardiographic predictor of short- and long-term adverse clinical outcomes in patients with acute myocardial infarction combined with new-onset right bundle branch block

**DOI:** 10.3389/fcvm.2023.1129235

**Published:** 2023-05-30

**Authors:** Jingchao Li, Haijia Yu, Luqian Cui, Huihui Song, Yingjie Chu, Shujuan Dong

**Affiliations:** ^1^ Department of Cardiology, Henan Provincial People’s Hospital, Zhengzhou, China; ^2^ Department of Emergency, Henan Provincial People’s Hospital, Zhengzhou, China; ^3^ Department of Cardiac Care Unit, Henan Provincial People’s Hospital, Zhengzhou, China

**Keywords:** acute myocardial infarction (AMI), right branch bundle block (RBBB), QRS duration, RV_6_-V_1_ interval, electrocardiographic (ECG), prognosis

## Abstract

**Background:**

A few studies have focused on electrocardiography (ECG) parameters correlating with clinical prognosis in patients with acute myocardial infarction (AMI) combined with new-onset right bundle branch block (RBBB).

**Objective:**

To assess the prognostic value of a new ECG parameter, namely, the ratio of QRS duration/RV_6_-V_1_ interval (QRS/RV_6_-V_1_), in patients with AMI combined with new-onset RBBB.

**Materials and methods:**

A total of 272 AMI patients combined with new-onset RBBB who received primary percutaneous coronary intervention (P-PCI) were retrospectively enrolled in the study. First, the patients were divided into survival group and non-survival group. Demographic, angiographic, and ECG characteristics were compared between the two groups. Receiver operating characteristic (ROC) curve was used to screen the best ECG parameter for predicting 1-year mortality. Second, the ratio of QRS/RV_6_-V_1_, a continuous variable, was converted to the high ratio group and low ratio group according to the optimal cutoff value point determined by the X-tile software. We compared the patient’s demographic, angiographic, and ECG characteristics, in-hospital major adverse cardiovascular events (MACE), and 1-year mortality between the two groups. Multivariate logistic and Cox regressions were used to evaluate whether the ratio of QRS/RV_6_-V_1_ was an independent prognostic factor of in-hospital MACE and 1-year mortality.

**Results:**

The ROC curve showed that the ratio of QRS/RV_6_-V_1_ had a higher value for predicting in-hospital MACE and 1-year mortality than the QRS duration, RV_6_-V_1_ interval, and RV_1_ interval. The patients in the high ratio group had significantly higher CK-MB peak and Killip class, lower ejection fraction (EF%), higher ratio of the left anterior (LAD) descending artery as infarct-related artery (IRA), and longer total ischemia time (TIT) than those in the low ratio group. The QRS duration was wider in the high ratio group than that in the low ratio group, whereas RV_6_-V_1_ was narrower in the high ratio group compared with that in the low ratio group. The in-hospital MACE rate (93.3% vs. 31.0%, *p* < 0.001) and 1-year mortality rate (86.7% vs. 13.2%, *p* < 0.001) in the high ratio group were higher than those in the low ratio group. The higher ratio of QRS/RV_6_-V_1_ was an independent predictor of in-hospital MACE (odds ratio, 8.55; 95% CI, 1.40–52.37; *p* = 0.02) after adjusting other confounders. Cox regression showed that the higher ratio of QRS/RV_6_-V_1_ predicted higher 1-year mortality of the patients with AMI combined with new-onset RBBB [hazard ratios (HR), 12.4; 95% CI, 7.26–21.22); *p* < 0.001] than the lower ratio of QRS/RV_6_-V_1_, and the HR still stayed at 2.21 even after a multivariable adjustment (HR, 2.21; 95% CI, 1.05–4.64); *p* = 0.037).

**Conclusion:**

According to the results of our study, the high ratio of QRS/RV_6_-V_1_ (>3.0) was a valuable predictor of short- and long-term adverse clinical outcomes in AMI patients combined with new-onset RBBB. The implications of the high ratio of QRS/RV_6_-V_1_ were severe ischemia and pseudo synchronization between bi-ventricle.

## Introduction

Electrocardiography (ECG), invented by Willem Einthoven nearly 120 years ago (1), is a standard diagnostic tool for acute myocardial infarction (AMI) with characteristics of easy availability and instantaneity. The previous studies demonstrated that many ECG parameters are associated with the prognosis of AMI (2). New-onset right bundle branch block (RBBB) is a well-recognized ECG marker predicting high mortality (3, 4) and an indication of emergency revascularization for AMI patients (5). Owing to the independent blood supply of the right bundle branch (RBB) from the left anterior descending artery (LAD), the incidence of new-onset RBBB in AMI is higher than that of the left branch bundle block (LBBB) (6). According to the differences of infarct-related artery (IRA) and total ischemic time (TIT), new-onset RBBB shows different characteristics on the ECG of AMI patients. Paul et al. proposed that qRBBB heralded more adverse prognosis than RBBB in AMI (7), which was consistent with the results of our previous study that showed atypical RBBB with worse prognosis than typical RBBB in AMI (8).

It is well known that QRS duration is associated with mortality in AMI patients (9). Prolonged QRS duration is a marker for an increased risk of AMI (10). Recently, another ECG parameter, RV_6_-V_1_ interpeak interval (RV_6_-V_1_ interval), was proposed for assessing the synchrony of bi-ventricle (11). It was used in the field of the left bundle branch (LBB) area pacing. Transition of a capture mode from nonselective LBB capture to selective LBB capture could result in a delay of right ventricular (RV) activation, because of the loss of RV depolarization via direct septal myocardial activation, decreasing the synchrony of bi-ventricle and increasing the RV_6_-V_1_ interval. Conversely, the RV_6_-V_1_ interval will decrease if the nonselective LBB capture converts to LV septal capture.

Myocardial ischemia and asynchrony of bi-ventricle are concurrent in AMI patients combined with new-onset RBBB. Theoretically, both QRS duration and RV_6_-V_1_ interval would change in this condition. To the best of our knowledge, the available ECG parameters which are unique for indicating the prognosis of AMI combined with new-onset RBBB are limited. In this study, we aimed to develop a new and reliable ECG parameter depending on the QRS duration and RV_6_-V_1_ interval, which could predict a short and/or long prognosis of AMI patients combined with new-onset RBBB.

## Materials and methods

### Study design and population

This retrospective cohort study was approved by the Ethics Committee of Henan Provincial People's Hospital (Approval No.: 2020-158). All patients aged ≥18 years who were hospitalized for AMI (12), routinely receiving primary percutaneous coronary intervention (PCI), combined with new-onset RBBB between 2007 August 1 and 2021 July 31 in our hospital were included. AMI was diagnosed based on the Fourth Universal Definition of Myocardial Infarction. Patients who suffered from preexisting RBBB, severe liver and kidney dysfunction, advanced malignant tumors, active bleeding, any other patterns of bundle branch block (BBB) other than RBBB pattern, left main (LM) or left circumflex (LCX) as IRA, and life expectancy of <1 year were excluded.

Demographics, prior comorbidities, data of auxiliary examination, and basic medication usage of the included patients were obtained from the EMR (electronic medical record) in our hospital. Since the function of the left ventricle would dynamically change during the whole process of treatment, we adopted the lowest ejection fraction (EF%) collected after the emergency PCI in our hospital to exclude the disturbance of operative intervention. There were two methods for grouping collected patients in our study. First, the patients were divided into survival and non-survival groups based on the primary endpoint for analyzing related variables. Second, we divided the ratio of QRS/RV_6_-V_1_ into low ratio and high ratio groups, using special statistical software, to track the difference of the patients’ prognosis between the two groups.

### Coronary angiography and intervention parameters

Coronary angiograms were re-analyzed by two independent cardiologists who were blinded to the clinical data. The IRA was defined as the coronary artery that was occluded or showed the most serious stenosis corresponding to ECG changes (13). TIT was the period from the symptom onset to balloon dilation for restoring blood flow. Diseased vessels were defined as ≥50% stenosis. Information on thrombolysis in myocardial infarction (TIMI) flow grade and interventional treatment were collected.

### ECG standard

The first ECG indicating new-onset RBBB upon arrival at the hospital was selected for further analysis. If the RBBB appeared after admission or presented on admission but was not recorded on an ECG performed within the previous 6 months, it was defined as new-onset RBBB. The paper speed of ECG conducted in our study was 25 mm/s, using Philips ECG Machine (the data was captured at a sample rate of 500 Hz). In addition, the morphology of RBBB in ECG was divided into typical and atypical RBBB. The ECG diagnostic criteria of AMI combined with new-onset RBBB and the difference between the two types of RBBB were as the same as our previous publication (8). Briefly, the wave forms of QRS in leads V_1_ or V_2_ demonstrated as rsR′ or M type were typical RBBB, whereas atypical RBBB manifested qR′ or R in leads V_1_ or V_2_ in ECG. If the new-onset RBBB disappeared before discharge, we identified it as transient RBBB. Another three ECG parameters indicating conduction time in the ventricle were included for the comparison between different groups. The methods of measuring important ECG parameters were as follows:

(1) QRS duration: vertical distance measured from the beginning of QRS to the final QRS component in the lead with the maximal ST deviation (14). When the J point could not be clearly distinguished, the QRS offset was defined as the point where a superimposed line was descending from the peak of the R-wave along 40% of its amplitude, between the R peak and the nadir of the ST-segment, intersecting with the PR segment baseline, based on the method proposed by Almer et al. (15) (Supplementary Figure S1). If the changes in the lead with maximum ST elevation were too large to measure, the closest adjacent lead was instead used (i.e., the lead with the second largest ST deviation) (Supplementary Figures S2 and S3).
(2) RV_1_ interval: vertical distance measured from the beginning of QRS to the peak of the dominant R-wave in lead V_1_.
(3) RV_6_-V_1_ interval: vertical distance measured from the R-wave peak in lead V_6_ to the R-wave peak in lead V_1_ during simultaneous recording of all 12 ECG leads (11) (Supplementary Figures S2 and S3).

Two experienced electrocardiologists, who were blinded to other clinical data, manually participated in the measurement of ECG intervals mentioned above. Particular methods were shown in Supplementary Figures S1–S3. The final data of the intervals were determined by an average originating from the results measured by the two electrocardiologists. Before the formal trial, these two electrocardiologists (one has a senior professional title) tried to separately test the RV_6_-V_1_ interval. The intraclass correlation coefficient (ICC) of the two results was 0.91, which indicated that the investigators’ evaluations were considered reliable.

### Endpoints and follow-up

In our study, the primary endpoint was 1-year all-cause mortality (1-year mortality), and the secondary endpoint was in-hospital major adverse cardiovascular events (MACE) including heart failure, cardiac shock, malignant ventricular arrhythmia, high-level atrioventricular block, and stroke. The patients were followed up to the events or, in the case of no event, up to 365 days. The follow-up work was conducted by a dedicated nursing team. The follow-up methods consisted of a telephone interview with the patients or with a close family member and/or having a review of the EMR.

### Statistical analysis

Continuous variables were presented as mean ± SD or median (interquartile range), as appropriate, and were compared by using the one-way analysis of variance of Kruskal–Wallis test. Discrete variables were described as counts (percentages) and were compared by using *χ*^2^ or Fisher's exact test. We used the X-tile software (16) (Yale University, New Haven, CT, United States) to determine the optimal cutoff value (3.0) of the QRS/RV_6_-V_1_ ratio which was dichotomized as high ratio and low ratio based on the cutoff point for further analysis.

Univariate logistic regression was used to screen the predictors for in-hospital MACE. Only variables with univariate *p*-values of <0.1 were further included in the multivariate model. We used multiple logistic regression to identify the independent predictors of in-hospital MACE. Odds ratios (OR) were used as a summary statistic. The 1-year mortality was analyzed by univariate and multivariate Cox regression adjusting for baseline factors that significantly predicted the outcomes. Hazard ratios (HR) were also used as the summary statistic. The receiver operating characteristic (ROC) curve was then used to evaluate the predicting value of the QRS/RV_6_-V_1_ interval for the 1-year mortality compared with the other three ECG parameters. All statistical analyses were performed using SPSS software version 25.0 and Rtools 4.2.

## Results

### Baseline and angiographic characteristics

**Figure 1:**
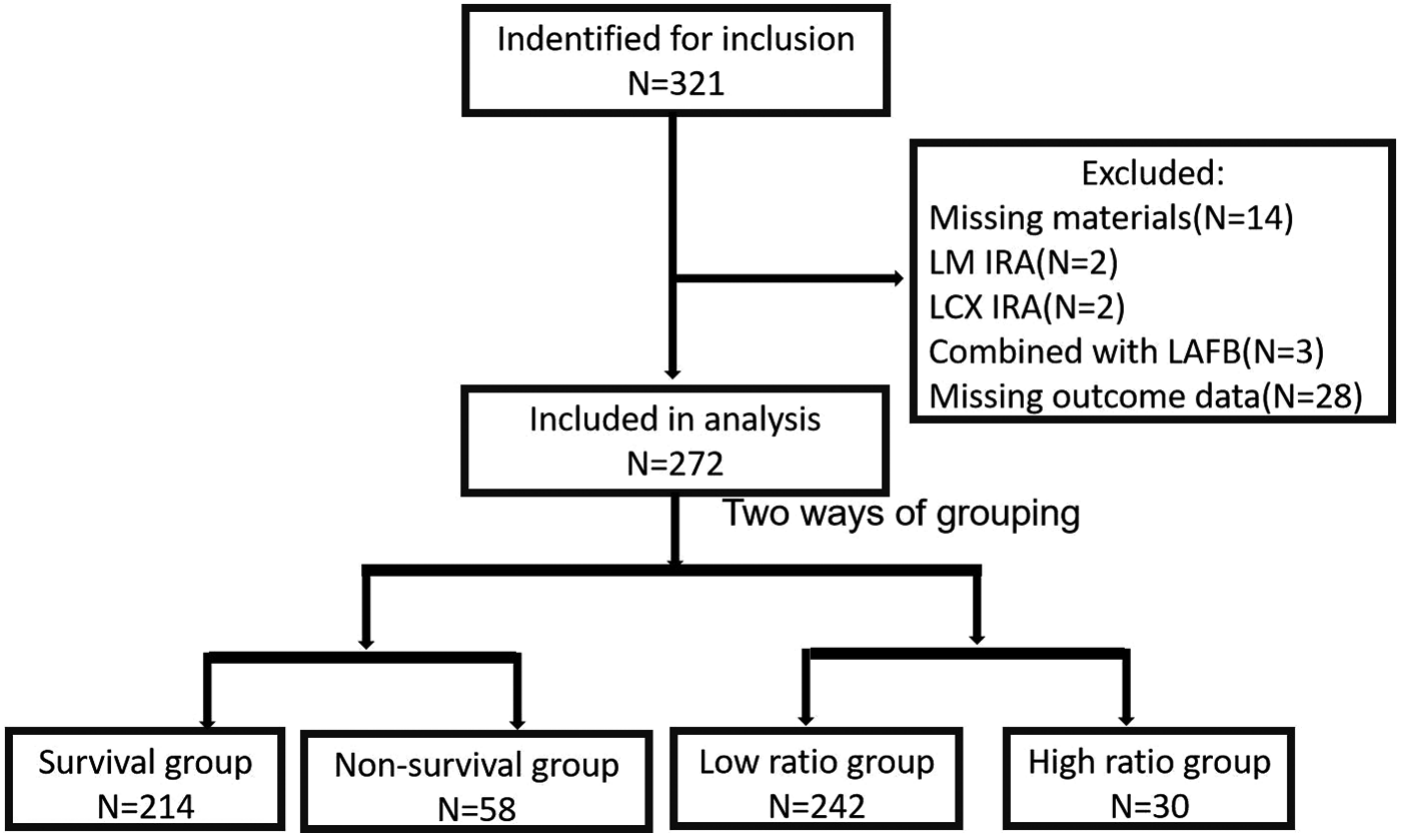
Flow chart summarizing the inclusion, exclusion, and grouping of the included patients.

In total, 321 patients (mean age: 65.89 ± 12.56 years) fulfilled the inclusion criteria. After excluding patients with incomplete data (*N* = 14), with LM (*N* = 2) and LCX (*N* = 2) as IRA, combined with left anterior fascicular block (LAFB, *N* = 3), missing primary or second outcome data (*N* = 28), 272 patients were included in the final analysis (Figure 1). All patients were divided into two groups, survival group (78.68%) and non-survival group (21.32%) based on the primary endpoint. The baseline, angiographic characteristics, and medicine usage were listed in Supplementary Table S1. The patients in the non-survival group were older and more likely to consume alcohol, had higher ratio of comorbidities such as diabetes and old myocardial infarction (MI), had a higher level of CK-MB peak value, and had worse heart function (expressed by lower EF% and higher ratio of Killip class ≥II) compared with the survival group. Based on the coronary angiography and interventional treatment data, patients in the non-survival group had longer TIT, higher ratio of LAD IRA, and multi-vessel lesion than those in the survival group. All patients received a standard medicine treatment based on the guideline of STEMI (5) and NSTEMI (17). More patients received diuretics and digoxin for relieving heart failure symptoms in the non-survival group compared with the survival group; however, there was no statistical difference between the two groups.

### ECG analysis

**Figure 2:**
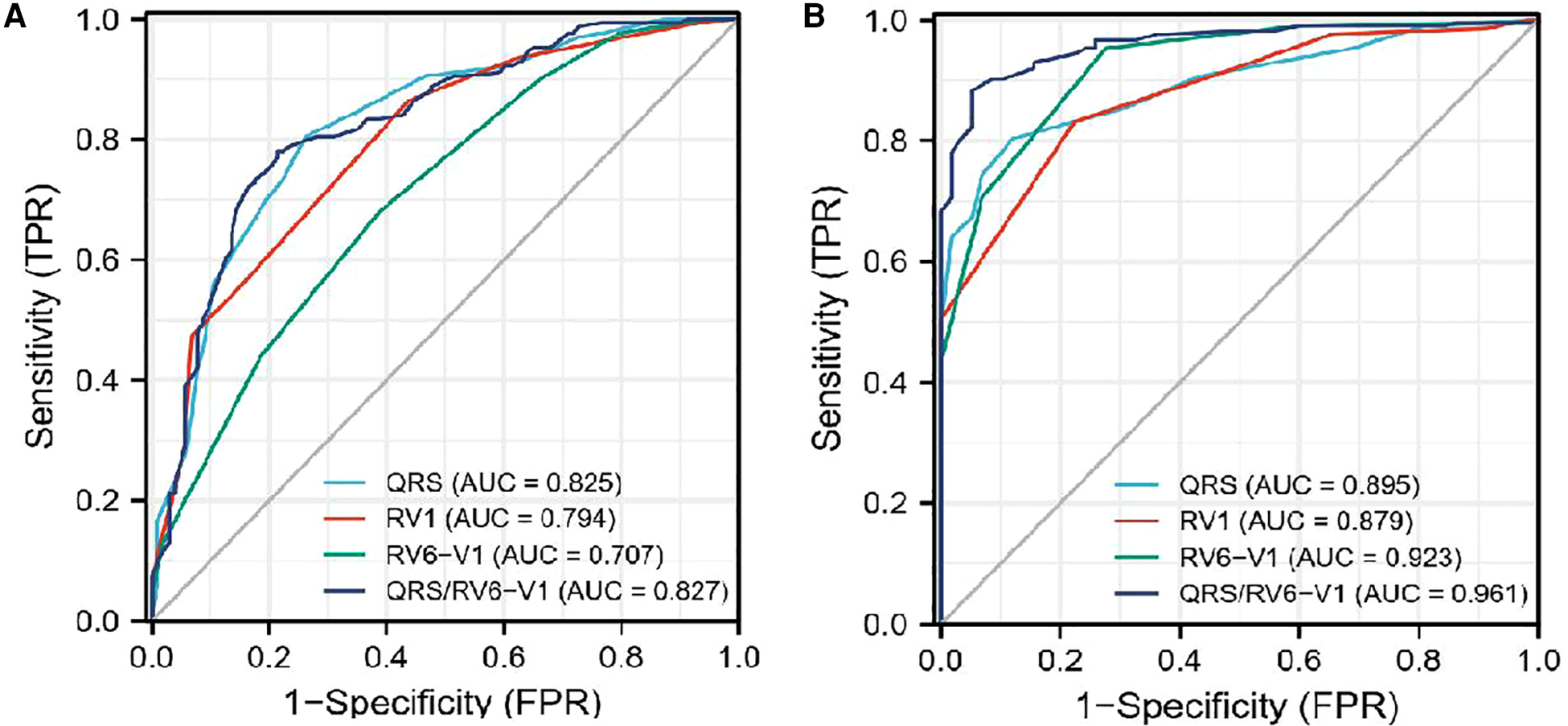
ROC curve analysis of the four different ECG parameters to predict prognosis. (**A**) ROC curves for in-hospital MACE. (**B**) ROC curves for 1-year mortality. ROC, Receiver operating characteristic; ECG, electrocardiography.

The associations between the primary endpoint and several special ECG parameters were compared between the survival group and the non-survival group (Table 1). A higher ratio of patients had atypical RBBB, but a lower ratio had transient RBBB in the non-survival group than those in the survival group. Patients in the non-survival group had wider QRS duration and RV_1_ interval but narrower RV_6_-V_1_ interval compared with those in the survival group. We included QRS/RV_6_-V_1_ ratio as a new ECG parameter to amplify the trend of change relating to QRS duration and RV_6_-V_1_. QRS/RV_6_-V_1_ ratio was higher in the non-survival group compared with that in the survival group. By ROC curve analysis, the AUC values of the QRS/RV_6_-V_1_ ratio were 0.827 (95% CI, 0.939–0.983) in predicting in-hospital MACE and 0.961 (95% CI, 0.773–0.876) in predicting a 1-year survival (Figure 2), which were higher than that of the QRS wave duration, RV_1_ interval, and RV_6_-V_1_ interval.

**Table 1:** ECG characteristics between the survival group and non-survival group.

	Survival group (*N* = 214)	Non-survival group (*N* = 58)	*p*-value
RBBB type (atypical, *n* %)	104 (48.6)	54 (93.1)	<0.001*
Transient RBBB, *n* (%)	86 (31.6)	4 (1.5)	<0.001*
STEMI, *n* (%)	171 (79.9)	38 (65.5)	0.021*
QRS duration (ms)	155.23 ± 23.3	190.26 ± 13.12	<0.001*
RV_1_ interval (ms)	113.43 ± 11.72	128.88 ± 6.94	<0.001*
RV_6_-V_1_ interval (ms)	76.1 ± 6.19	64.31 ± 5.33	<0.001*
Ratio (QRS/RV_6_-V_1_)	2.06 ± 0.38	2.98 ± 0.34	<0.001*

ECG, electrocardiography; RBBB, right branch bundle block; STEMI, ST-segment elevation myocardial infarction.

^*^ Statistically significant results.

### Comparison of the low ratio and high ratio group of QRS/RV_6_-V_1_

**Figure 3:**
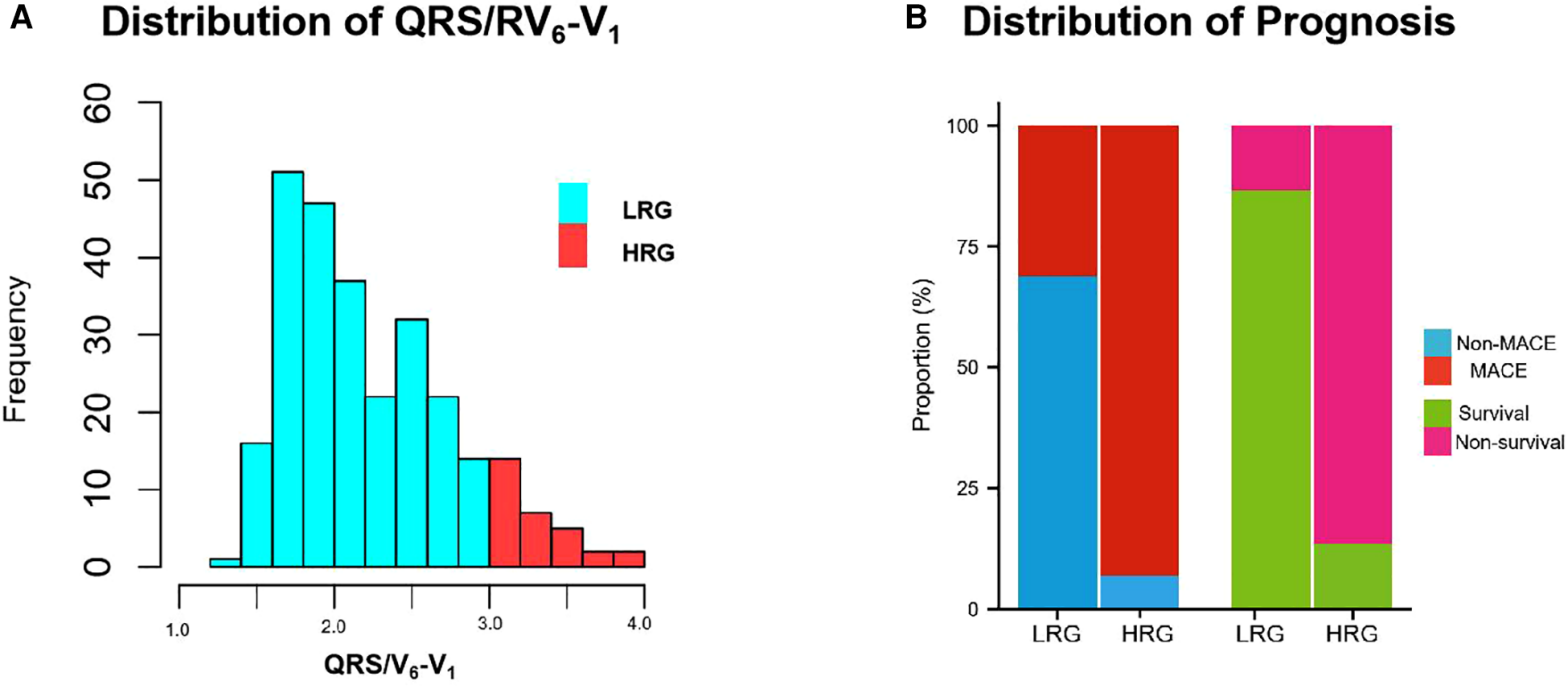
Frequency-values histogram of QRS/RV_6_-V_1_ distribution and prognostic difference between LRG and HRG. (**A**) Population distribution in LRG and HRG. (**B**) The difference of prognostic distribution between LRG and HRG, including MACE and 1-year mortality. MACE, major adverse cardiovascular events; LRG,low ratio group; HRG,high ratio group.

According to the cutoff value point (3.0) of QRS/RV_6_-V_1_ ratio by X-tile software, all patients included in our study were divided into the low ratio group (242 patients, 88.97%) and the high ratio group (30 patients, 11.03%) (Figure 3A). The demographics, prior comorbidities, data of auxiliary examination, coronary angiography and intervention information, ECG characteristics, and prognosis were compared between the two groups. Results were presented in Supplementary Tables S2 and S3. The patients in the high ratio group were older than those in the low ratio group. More patients suffered from diabetes and old MI in the high ratio group. Higher CK-MB peak level and Killip class and lower EF% were observed in the high ratio group than those in the low ratio group. The patients in the high ratio group had a higher ratio of LAD as IRA, multi-vessel disease, and longer TIT compared with those in the low ratio group. The in-hospital MACE (93.3% vs. 31.0%, *p* < 0.01) and 1-year mortality (86.7% vs. 13.2%, *p* < 0.01) in the high ratio group were higher than those in the low ratio group (Figure 3B). All the differences mentioned above had statistical significance.

Regarding the ECG parameters, the high ratio group had a higher proportion of atypical RBBB (83.3% vs. 55.0%, *p* = 0.003) and lower proportion of transient RBBB (6.7% vs. 36.4%, *p* = 0.001) compared with the low ratio group. The QRS duration (198.83 ± 10.72 vs. 158.22 ± 23.59, *p* < 0.001) and RV_1_ interval (128.83 ± 8.579 vs. 115.23 ± 12.20, *p* < 0.001) were longer, whereas the RV_6_-V_1_ interval (60.50 ± 4.80 vs. 75.21 ± 6.34, *p* < 0.001) was shorter in the high ratio group than that in the low ratio group.

### Prognostic value of QRS/RV_6_-V_1_

**Figure 4:**
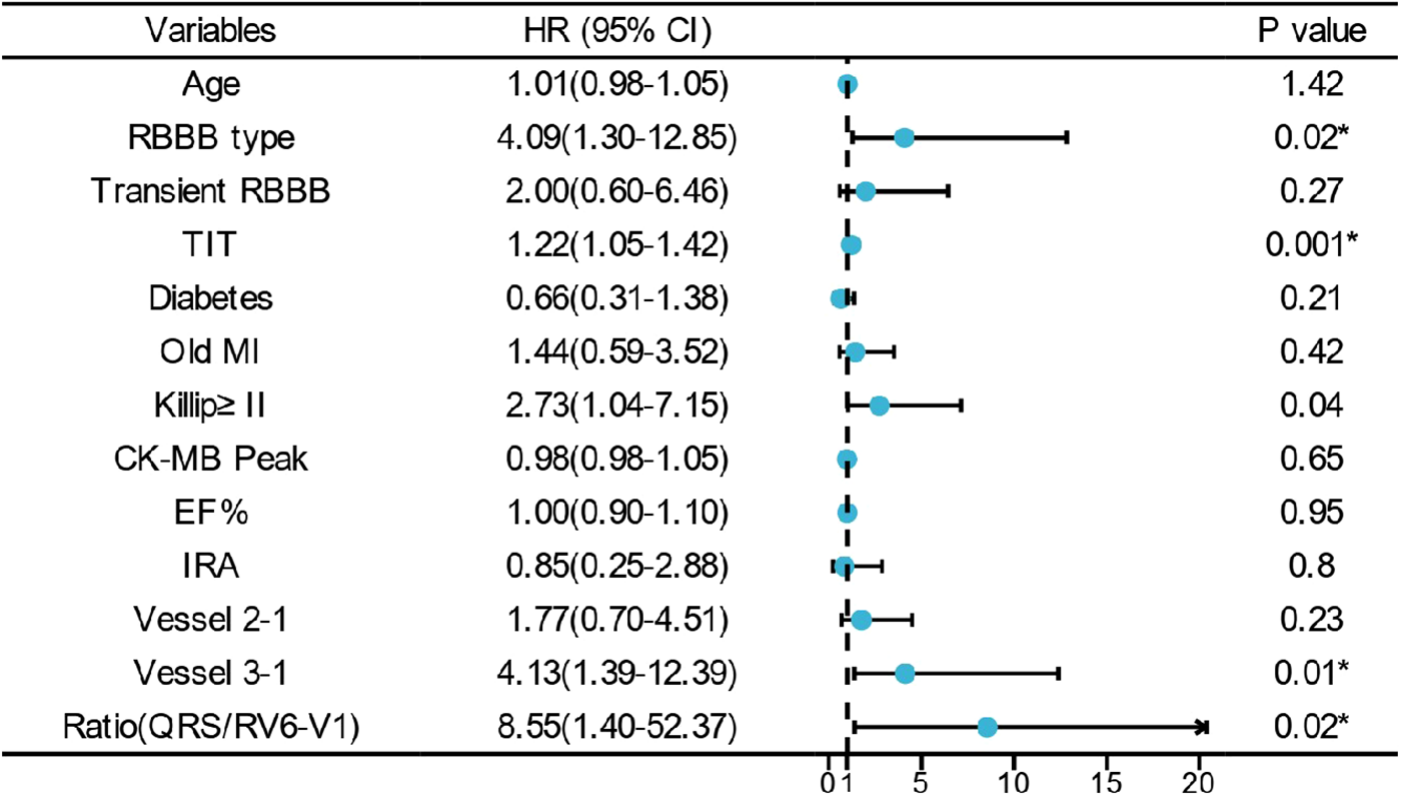
Multivariate logistic regression analysis of in-hospital MACE. * indicates that the variable was an independent predictor of in-hospital MACE. RBBB, right bundle branch block; TIT, total ischemic time; MI, myocardial infarction; EF, ejection fraction.

All in all, 103 cases of in-hospital MACE occurred in the cohort [75 cases in the low ratio group and 28 cases in the high ratio group (31.0% vs. 93.3%, *p* < 0.01)]. Univariate and multivariate logistic regression analyses were used to identify the association between QRS/RV_6_-V_1_ ratio and in-hospital MACE. Totally, 13 predictors such as age, RBBB type, transient RBBB, TIT, and so on were screened out as the in-hospital MACE predictors. After adjusting for confounders, the high ratio of QRS/RV_6_-V_1_ [OR, 8.546; 95% CI, 1.395–52.367); *p* = 0.02] was still an independent predictor of in-hospital MACE together with RBBB type, TIT, Killip class of ≥II, and multi-vessel disease (Figure 4).

**Figure 5:**
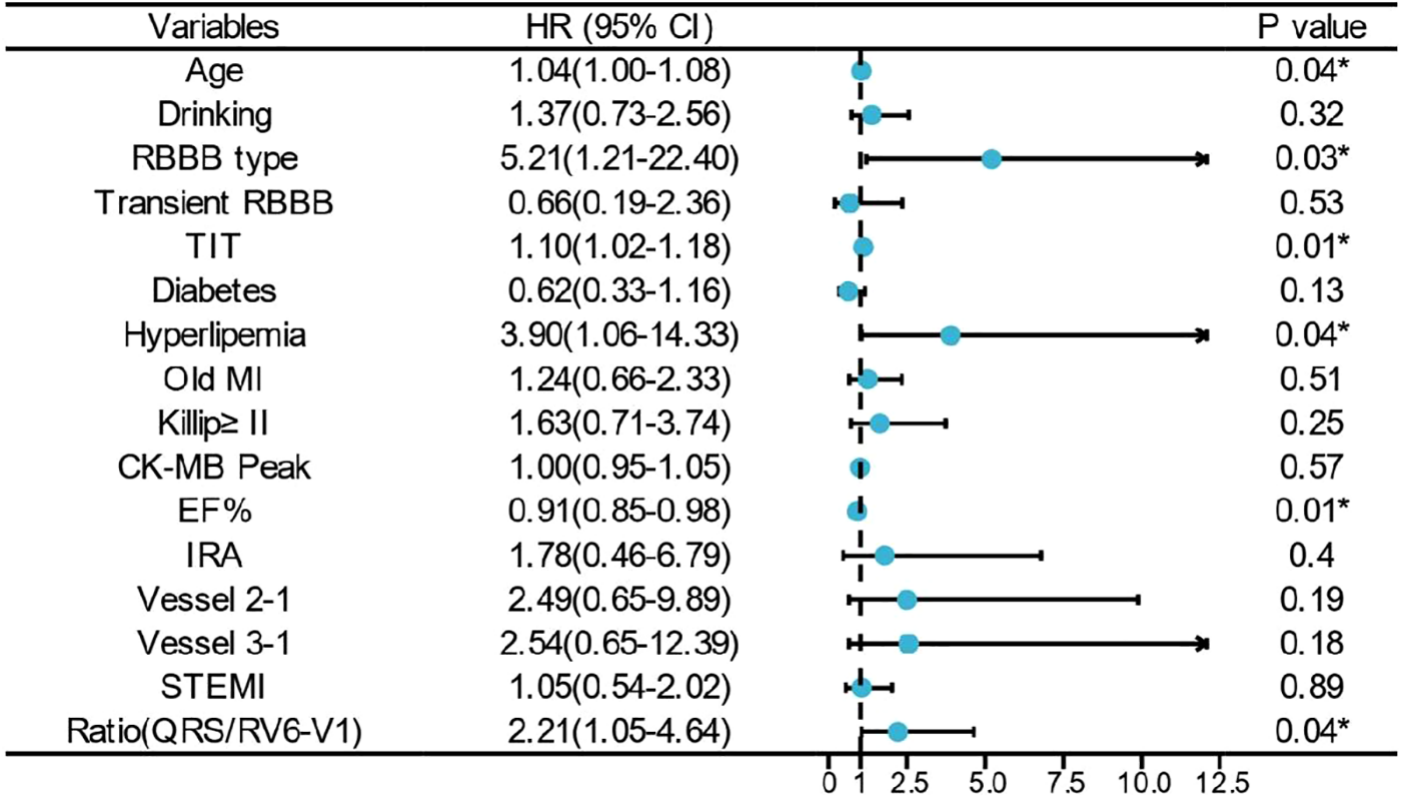
Multivariate Cox regression analysis of 1-year mortality. * indicates that the variable was an independent predictor of 1-year mortality. RBBB, right bundle branch block; TIT, total ischemic time; MI, myocardial infarction; EF, ejection fraction.

**Figure 6:**
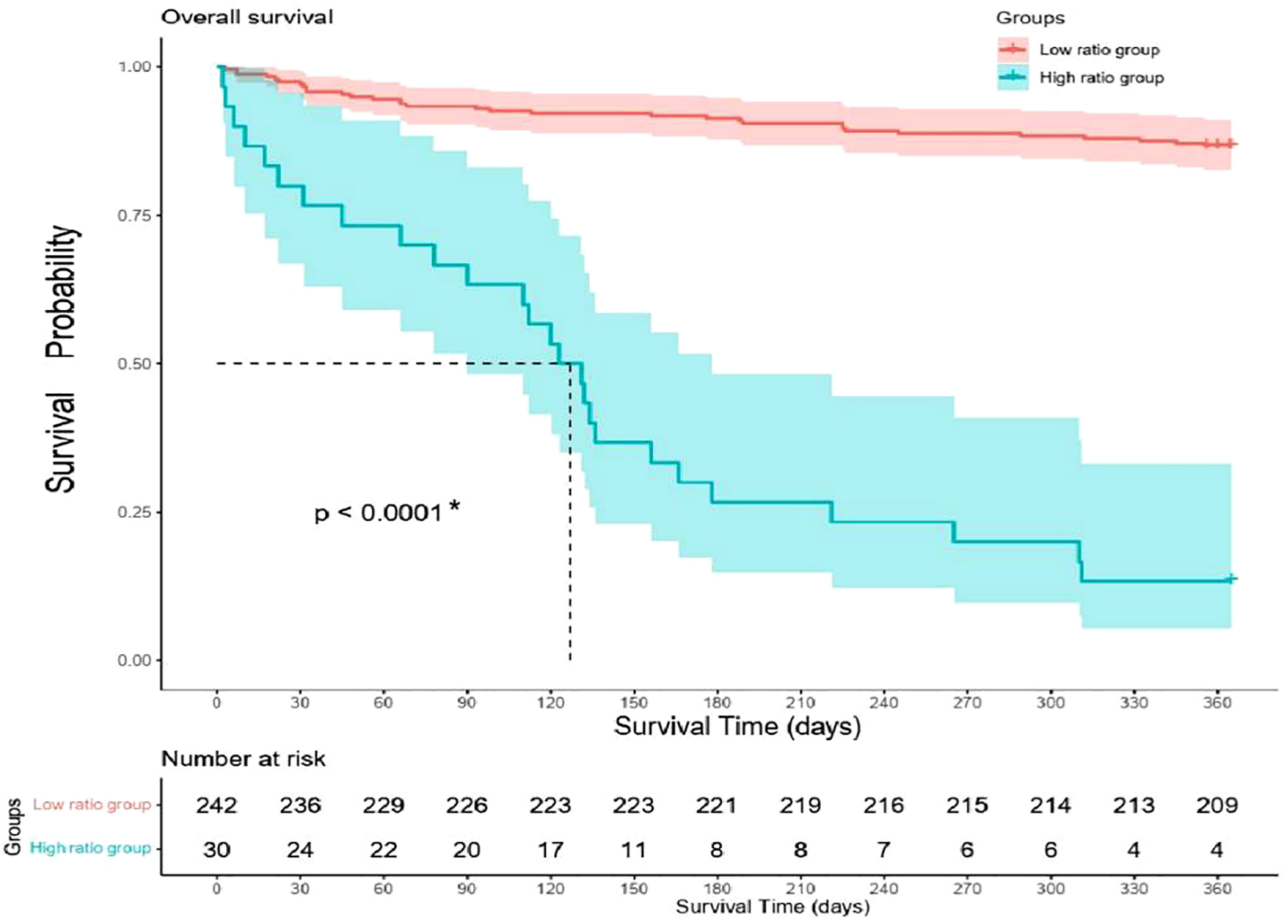
Kaplan–Meier curves of 1-year mortality difference between high ratio group and low ratio group.

All the patients were followed up for 1 year unless the primary endpoint occurred before that time. The Cox regression showed that the high ratio of QRS/RV_6_-V_1_ predicted the 1-year mortality of AMI patients combined with new-onset RBBB (13.2% vs. 86.7%; HR, 12.4; 95% CI, 7.26–21.22); *p* < 0.001), which still had a statistical value after multivariable adjustment (HR, 2.21; 95% CI, 1.05–1.4.64); *p* = 0.037) (Figure 5). The Kaplan–Meier analysis showed that more patients died at the 1-year endpoint in the high ratio group than that in the low ratio group of QRS/RV_6_-V_1_ (Figure 6).

## Discussion

AMI is one of the major causes of mortality worldwide (18). Nowadays, the most efficient treatment of AMI is primary PCI (19). The principle of primary PCI emphasizes on opening the IRA early, completely, and continuously (5). Therefore, early diagnosis of AMI is crucial. ECG is the most easily available of all the auxiliary tests for AMI. New-onset RBBB has been included as an early indication of revascularization since 2017 in ESC guidelines for STEMI, thereby highlighting its value in primary revascularization (5). The prognostic value of new-onset RBBB in AMI has been demonstrated (4, 8, 20). Previous studies even proposed that the mortality of patients with AMI combined with RBBB was higher than that combined with LBBB (21), especially atypical RBBB (7, 8). Several ECG indices predicting the adverse prognosis of AMI aside from BBB, such as the QRS duration (9, 15), J wave and T wave alternations, and so on, existed (23). However, to the best of our knowledge, none of them was special for AMI patients combined with new-onset RBBB.

QRS duration represents the time of ventricular depolarization, 0 associating with the prognosis of AMI patients. RV_6_-V_1_ is a new ECG parameter, which has been recently proposed, predicting the synchrony of bi-ventricle. Therefore, we proposed a novel ECG parameter depending on the two indices, namely, QRS/RV_6_-V_1_ ratio, to predict the clinical prognosis of AMI patients combined with new-onset RBBB especially in this study.

The main findings of our study were as follows: (1) The high ratio of QRS/RV_6_-V_1_ implied a serious ischemia of the left ventricle and pseudo synchronization of bi-ventricle. (2) The high ratio of QRS/RV_6_-V_1_ was a novel independent predictor of a short- and long prognosis of AMI patients combined with new-onset RBBB.

### Comparison of the survival and non-survival groups in AMI patients combined with new-onset RBBB

We retrospectively analyzed 272 cases of patients who fulfilled the inclusion criteria of this research and divided them into the survival group and the non-survival group first based on the primary endpoint. The 1-year mortality rate of AMI combined with new-onset RBBB was 21.32%, which was consistent with previous studies (7, 22). In this study, the patients in the non-survival group had a higher level of CK-MB peak and lower EF% and a higher ratio of Killip class of ≥II than those in the survival group, which denoted a serious damage of the myocardium and cardiac function in the non-survival group. The serious damage may be associated with longer TIT, higher ratio of LAD IRA, and multi-vessel lesions representing a serious and larger area of ischemia and necrosis of the myocardium. The results of our included patients mentioned above, a subset of AMI patients, were similar with that of the previous studies of the whole AMI population (23, 24). Although the difference of the basic medications did not have a statistical significance between the survival group and the non-survival group, we could also detect that more patients in the non-survival group used medications for heart failure, which conveyed a serious damage of heart function. However, the differences of several ECG parameters between the two groups were the important results for us in this study.

Previous articles demonstrated that the QRS duration was associated with the time of ventricular depolarization (15), and the RV_1_ interval (a portion of QRS wave in lead V_1_) represented the excitation time of the right ventricle and RV_6_-V_1_ interval (11) were on behalf of the non-synchronous conduction between the left and right ventricles. Apart from the functional disorder of the BBB, QRS duration could also be affected by ischemia and hyperkalemia in AMI patients. Previous studies demonstrated that hyperkalemia was common in AMI patients, associating with higher risks for adverse outcomes (25). Therefore, the QRS duration and RV_1_ interval were longer in the non-survival group than those in the survival group in this study, which was not only due to RBBB but also due to ischemia and potential problem of hyperkalemia. To our surprise, the RV_6_-V_1_ interval was shorter in the non-survival group, which indicated that the synchrony of bi-ventricle ameliorated. Due to the lengthening of QRS wave and shortening of RV_6_-V_1_ interval, the ratio of QRS/RV_6_-V_1_ was quoted as a new ECG parameter to amplify the difference of the two parameters in our study. The ROC curve demonstrated that the QRS/RV_6_-V_1_ ratio had a higher predicted value of the in-hospital MACE and 1-year mortality than the other three ECG parameters, which enhanced our confidence for paying attention to the new ECG parameter. To explore the value of QRS/RV_6_-V_1_ ratio in predicting the short- and long-term prognosis in AMI patients combined with new-onset RBBB, we converted it to a categorical variable, namely, the high ratio group and the low ratio group, based on the optimal cutoff point screened by X-tile software for further analysis.

### Comparison of the high ratio group and the low ratio group of QRS/RV_6_-V_1_

Viktor et al. proposed the concept of “ischemic QRS,” presenting a terminal QRS distortion and resultant QRS prolongation (14). Wong et al. reported that QRS duration indicated a 30-day mortality independently in patients with anterior AMI, either with or without RBBB (26). Therefore, in our study, a wider QRS duration in the high ratio group may be associated with longer TIT, multi-vessel diseases, higher CK-MB peak, and older patients which indicated a serious ischemia and weaker resistant ability of ischemia. However, the cause of this shortened RV_6_-V_1_ interval in the high ratio group than that in the lower ratio group needs further discussion. Did this phenomenon represent an improved synchrony of in the high ratio group?

**Figure 7:**
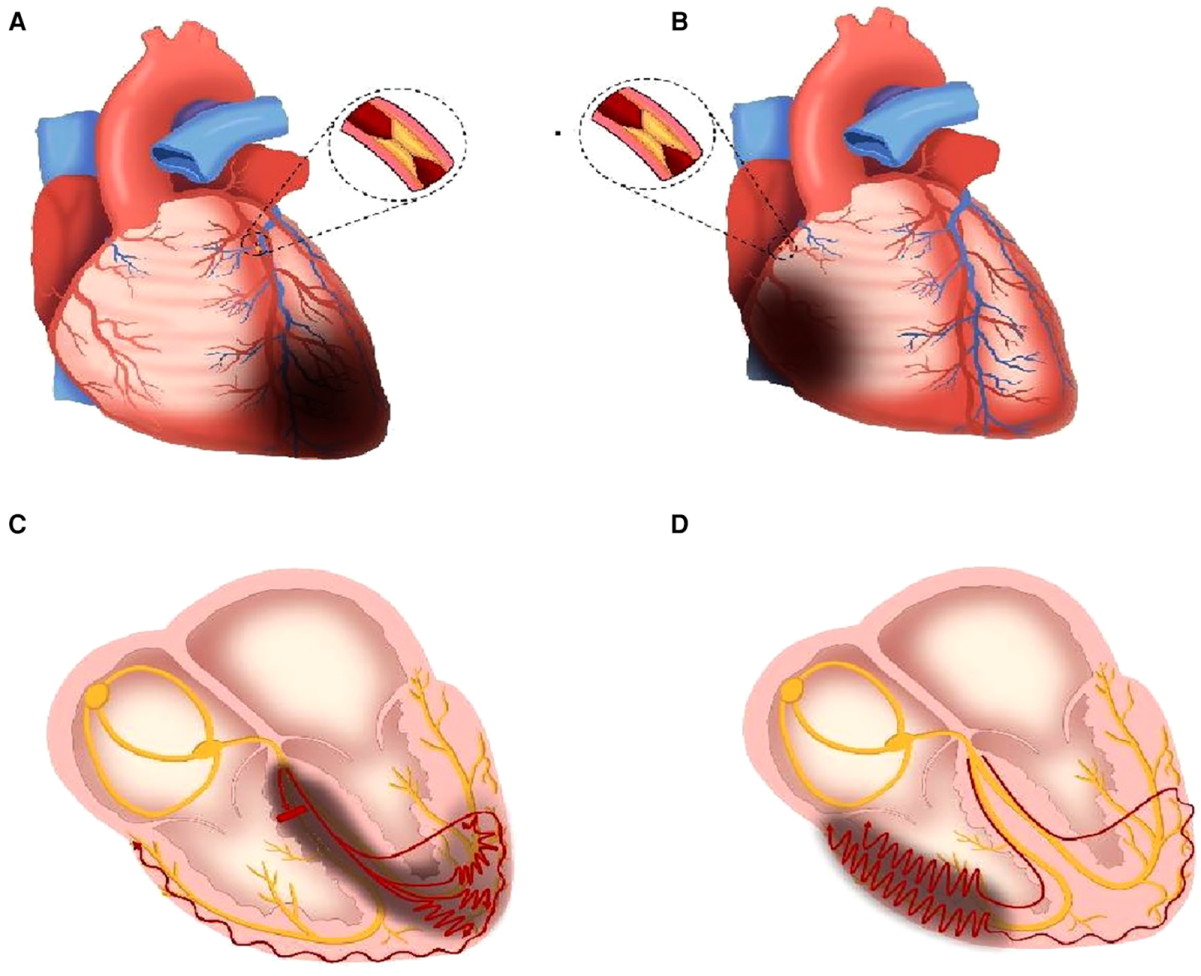
Mechanism schematic diagram of the new-onset RBBB in AMI. (**A**) LAD proximal occlusion leads to ischemia and necrosis of the interventricular septum, partial left ventricle, and cardiac apex. (**B**) RCA proximal occlusion leads to ischemia and necrosis of the partial right ventricle. (**C**) RBB is injured (short, thick, red line means blocked conduction) due to ischemia, and the speed of electrical conduction in partial left ventricular myocytes slows down (red curves). (**D**) The speed of electrical conduction in partial right ventricular myocytes slows down (red curves). The red curves in (**C,D**) mean the slow conduction in the ventricles, and more circuitous means slower. The dark gray areas mean ischemia and necrosis. RBB, right branch bundle; LAD, left anterior descending branch; RCA, right coronary artery.

Anatomically, the LAD offers blood supply for the RBB. A previous research proposed that the causes of new RBBB onset in AMI were due to the proximal occlusion of LAD, running through the interventricular septum (Figure 7A), or ischemia and dilation of the right ventricle due to proximal occlusion of RCA (Figure 7B) (8). In a study focused on the mechanical implication of LBBB using cardiovascular magnetic resonance (CMR), Baritussio et al. reported that 31% of LBBB patients suffered from ischemic heart disease, and 47% experienced LBBB-related septal asynchrony, which indicated that not only branch bundle damage but also ventricular ischemia could result in new-onset BBB in ECG (27).

In the high ratio group, more IRA was LAD compared with that in the low ratio group (Supplementary Table S1). When the occlusion occurred in the proximal end of the LAD, the RBB may be injured due to the reduction of blood supply, and the myocardium in the interventricular septum, left ventricle, and cardiac apex could suffer from ischemia or necrosis (Figure 7A). Although the right ventricular depolarization was delayed due to the right branch bundle destroyed, the conduction speed in the left ventricle also slowed because of the ischemia of multiple areas in the left ventricle (Figure 7C). When the conduction velocity decreased in the left and right ventricles simultaneously, the time interval (RV_6_-V_1_ interval) would be narrowed. Wider QRS duration combined with shorter RV_6_-V_1_ interval, leading to the increase of QRS/RV_6_-V_1_ ratio, denoted a serious ischemia and pseudo synchronization between the left and right ventricles.

On the other hand, IRA was RCA in 36.5% of patients in the low ratio group, but none in the high ratio group (Supplementary Table S2). When proximal RCA occluded, conduction delay would occur in the right ventricle due to right ventricular ischemia (Figures 7B,D). The level of ischemia induced by proximal RCA occlusion is often milder than that induced by proximal LAD; therefore, the QRS duration would be shorter when RCA occluded than when LAD occluded. The QRS duration which was shorter in the low ratio group than that in the high ratio group may be attributed to the coronary anatomy and IRA mentioned above. Moreover, the ratios of the transient right bundle branch and typical right bundle branch were higher in the low ratio group than those in high ratio group, thus indicating a mild injury of the myocardium (7, 28). In addition, the results such as lower CK-MB peak, higher EF%, shorter TIT, lower Killip class, and lower ratio of LAD IRA in our study supported that the delayed conduction caused by myocardial ischemia, especially the left ventricle, was milder in the low ratio group than that in the high ratio group. The mechanisms associated with the delayed depolarization of the right ventricle were dysfunction of RBB or ischemia of the right ventricular myocardium in the low ratio group, whereas the conduction speed in the left ventricle was almost normal or slightly decreased. Therefore, the asynchronization between the left and right ventricles would be more obvious, which led to longer RV_6_-V_1_ interval on this condition. Mild ischemia and obvious asynchronization were the main reasons of the low QRS/RV_6_-V_1_ ratio.

### The predicted value of QRS/RV_6_-V_1_ ratio

As an ECG parameter, QRS was considered a predictor of left ventricular systolic dysfunction after a first STEMI by Fabiszak et al. (29). Bernikova et al. reported that the QRS duration prolonged after LAD occlusion (30). The QRS duration also had a special prognostic significance in NSTEMI patients (10). The prognostic value of QRS duration was also demonstrated in our study and was consistent with the previous studies. However, its value in predicting the 1-year mortality was less than the QRS/RV_6_-V_1_ ratio, which was proven by the ROC curve. Although the RV_6_-V_1_ interval has been used as a novel criterion for the diagnosis of LBB capture due to its value in representing synchrony of bi-ventricle, it has never been used in the domain of AMI, especially in the subset of patients with new-onset RBBB.

To the best of our knowledge, ours is the first study to combine the two ECG parameters to estimate its value in predicting short- and long-term adverse clinical outcomes in AMI patients combined with new-onset RBBB. After adjusting for relevant confounder, the high ratio of QRS/RV_6_-V_1_ was an independent predictor of in-hospital MACE together with RBBB type, TIT, Killip class, and multi-vessel disease (vessel 3-1 in the Figures 4 and 5) which indicated a serious damage of the myocardium and heart function. Adding the effect of time on prognosis, the QRS/RV_6_-V_1_ ratio could also predict the 1-year mortality of patients with AMI combined with new-onset RBBB after a multivariate adjustment. However, age, RBBB type, hyperlipidemia, and multi-vessel disease were also independent predictors of the 1-year mortality. Atypical RBBB which conveyed a larger area of infarction and was associated with a prognosis had been demonstrated by our previous study (8). Recently, an article reported that hyperlipidemia hampered the repairs of AMI-induced cardiac injury (31), and multiple studies proposed that hyperlipidemia and multi-vessel disease were associated with an adverse prognosis of AMI patient, which were also applicable in AMI patients combined with RBBB, consisting with the results of our study. In summary, we can consider to quote this new ECG parameter as a new indicator for prognosis in AMI patients combined with new-onset RBBB in clinic[28], which will help physicians judge high-risk patients.

### Limitation

There were several limitations in our study. First, this was a retrospective study and included only a small population from a single center. Second, although we used two grouping methods, the sample difference between groups was evident that may have affected the statistical analysis. Third, the ECG characteristics likely changed dynamically; the first ECG presented with new-onset RBBB upon arrival at the hospital may not be the optimum one representing the highest QRS/RV_6_-V_1_ ratio, which would influence the results. Fourth, the underlying mechanisms associated with the change of QRS/RV_6_-V_1_ ratio were only concluded by the statistical analysis of the clinical data. CMR and mapping of cardiac potential are further required to demonstrate the ischemic area and conduction speed of the myocardium. A prospective study to verify the diagnostic applicability of the new ECG parameter is under investigation, and digital ECG and automated measurement of time interval in ECG will be used in the subsequent studies.

## Conclusion

In this study, we proposed a new ECG parameter, namely, the QRS/RV_6_-V_1_ ratio, as a predictor of short- and long-term adverse clinical outcomes in AMI patients combined with new-onset RBBB. A higher QRS/RV_6_-V_1_ ratio indicates a serious ischemia and pseudo synchronization between the left and right ventricles, which should be given more attention in emergency treatment and longer duration of long-term follow-up.

## Data Availability

The raw data supporting the conclusions of this article will be made available by the authors, without undue reservation.
