# Supplementary material for: The ratio of QRS/RV6-V1: a new electrocardiographic predictor of short- and long-term adverse clinical outcomes in patients with acute myocardial infarction combined with new-onset right bundle branch block
Source: Front Cardiovasc Med. 2023 May 30;10:1129235. doi: 10.3389/fcvm.2023.1129235 (PMC10267865; doi:10.3389/fcvm.2023.1129235)
Supplement: Supplementary file 1 [file Datasheet1.docx]

**Supplement**

**TABLE S1.**.Baseline characteristics and coronary related information between Survival group and Non-survival group.

**TABLE S2**|Baseline characteristics and coronary related information between High ratio group and Low ratio group.

**TABLE S3**|The differences of ECG characteristic between High ratio group and Low ratio group.

**FIGURE S1**| Illustration of how the QRS offset is defined in the absence of a clear J point.

**FIGRUE S2|**The methods of measuring important ECG parameters,including QRS duration and RV6-V1 interval,in the circumstance of inferior wall myocardial infarction.

**FIGRUE S3|**The methods of measuring important ECG parameters,including QRS duration and RV6-V1 interval,in the circumstance of anterior wall myocardial infarction.

**The Ratio of QRS/RV_6_-V_1_—A New Electrocardiographic Predictor of Short- and Long-Term Adverse Clinical Outcomes in Patients with Acute Myocardial Infarction Combined with New Onset Right Bundle Branch Block**

**TABLE S1**.Baseline characteristics and coronary related information between Survival group and Non-survival group.

|  | Survival group  (N= 214 ) | Non-Survival group  (N= 58 ) | P-value |
| --- | --- | --- | --- |
| Demographics |  |  |  |
| Sex(male,n%) | 159(78.7) | 43(74.1) | 0.980 |
| Age,years | 63.6±12.5 | 74.3±8.5 | <0.001* |
| BMI(kg/㎡) | 28.1±2.8 | 28.7±2.7 | 0.867 |
| Hobby |  |  |  |
| Smoking,n(%) | 148(69.2) | 43(74.1) | 0.462 |
| Drinking,n(%) | 122(57.1) | 43(74.1) | 0.018* |
| Comorbidities |  |  |  |
| Hypertension,n(%) | 141(65.9) | 39(67.2) | 0.847 |
| Diabetes,n(%) | 87(40.7) | 35(60.3) | 0.007* |
| Hyperlipemia,n(%) | 184(86.0) | 55(94.8) | 0.067 |
| Old MI,n(%) | 47(22.0) | 30(51.7) | <0.001* |
| Examination |  |  |  |
| CK-MB Peak,(U/L) | 256.9±90.8 | 411.7±106.1 | <0.001* |
| EF% | 47.7±5.8 | 36.5±5.4 | <0.001* |
| Killip class≥ II,n(%) | 43(20.1) | 46(79.3) | <0.001* |
| Coronary Intervention |  |  |  |
| TIT,(h) | 7.9±3.36 | 14.69±5.0 | <0.001* |
| IRA |  |  | <0.001* |
| LAD,n(%) | 133(62.1) | 53(91.4) |  |
| RCA,n(%) | 81(31.9) | 5(8.6) |  |
| Diseased vessels |  |  | <0.001* |
| Vessel-1,n(%) | 118(55.1) | 3(5.2) |  |
| Vessel-2,n(%) | 49(22.9) | 24(41.4) |  |
| Vessel-3,n(%) | 47(22.0) | 31(53.4) |  |
| TIMI 0/1,n(%) | 185(86.0) | 48(82.8) | 0.677 |
| PCI,n(%) | 199(93.0) | 53(91.4) |  |
| Medication |  |  |  |
| [Aspirin](javascript:;),n(%) | 208(97.2) | 54(93.1) | 0.142 |
| Statin,n(%) | 202(94.4) | 54(93.1) | 0.753 |
| Clopidogrel/Ticagrelor,n(%) | 209(97.7) | 54(93.1) | 0.101 |
| ACEI/ARB,n(%) | 159(74.3) | 37(63.8) | 0.114 |
| ARNI,n(%) | 24(11.2) | 8(13.8) | 0.589 |
| Nitrates, n (%) | 69(32.2) | 17(29.3) | 0.670 |
| Beta blocks,n(%) | 102(47.7) | 23(39.7) | 0.278 |
| Diuretics, n (%) | 42(19.6) | 18(31.0) | 0.063 |
| Digoxin ,n(%) | 14(6.5) | 7(12.1) | 0.132 |

TIT, total ischemia time; MI, myocardial infarction; IRA, infarct related artery; LAD, left anterior descending artery; RCA, right coronary artery;PCI,percutaneous coronary intervention;TIMI,thrombolysis in myocardial infarction;ACEI, angiotensin-converting-enzyme inhibitors; ARB, angiotensin II receptor blockers; ARNI, angiotensin receptor neprilysin inhibitor. Statistically significant results were highlighted as *.

**TABLE S2**|Baseline characteristics and coronary related information between High ratio group and Low ratio group.

|  | Low Ratio group  (QRS/RV_6_-V_1_≤3.0）  (n= 242 ) | High Ratio Group  (QRS/RV_6_-V_1_＞3.0）  (n= 30 ) | P-value |
| --- | --- | --- | --- |
| Demographics |  |  |  |
| Sex(male,n%) | 180(74.4) | 22(73.3) | 0.902 |
| Age,years | 64.6±12.6 | 76.3±6.2 | <0.001* |
| BMI(kg/㎡) | 28.2±2.8 | 28.4±2.8 | 0.917 |
| Hobby |  |  |  |
| Smoking,n(%) | 170(70.2) | 21(70.0) | 0.978 |
| Drinking,n(%) | 144(59.5) | 21(70.0) | 0.267 |
| Comorbidities |  |  |  |
| Hypertension,n(%) | 163(67.4) | 17(56.7) | 0.243 |
| Diabetes,n(%) | 102(42.1) | 20(66.7) | 0.011* |
| Hyperlipemia,n(%) | 211(87.2) | 28(93.3) | 0.331 |
| Old MI,n(%) | 63(26.0) | 14(47.6) | 0.018* |
| Examination |  |  |  |
| CK-MB Peak,(U/L) | 268.5±97.5 | 462.0±83.6 | <0.001* |
| EF% | 46.6±6.5 | 35.0±4.9 | <0.001* |
| Killip≥ II,n(%) | 62(25.6) | 27(90.0) | <0.001* |
| Coronary Intervention |  |  |  |
| TIT,(h) | 8.6±4.1 | 15.1±5.1 | <0.001* |
| IRA |  |  | <0.001* |
| LAD,n(%) | 165(64.5) | 30(100) |  |
| RCA,n(%) | 86(35.5) | 0 |  |
| Diseased vessels |  |  | <0.001* |
| Vessel-1,n(%) | 119(49.2) | 2(6.7) |  |
| Vessel-2,n(%) | 59(24.4) | 14(46.7) |  |
| Vessel-3,n(%) | 64(26.4) | 14(46.7) |  |
| TIMI 0/1,n(%) | 210(86.8) | 23(76.7) | 0.879 |
| PCI,n(%) | 224(92.6) | 28(93.3) | 0.136 |
| Prognosis |  |  |  |
| In-hospital MACE,n(%) | 75（31.0） | 28（93.3） | <0.001* |
| 1-year Mortality,n(%) | 32（13.2） | 26（86.7） | <0.001* |

TIT, total ischemia time; MI, myocardial infarction; IRA, infarct related artery; LAD, left anterior descending artery; RCA, right coronary artery;PCI,percutaneous coronary intervention;TIMI,thrombolysis in myocardial infarction;MACE,major adverse cardiovascular events.Statistically significant results were highlighted as *.

**TABLE S3**|ECG characteristic between High ratio group and Low ratio group.

|  | Low Ratio Group  (QRS/RV6-V1≤3.0）  (n=242) | High Ratio group  (QRS/RV6-V1＞3.0）  (n=30) | P-value |
| --- | --- | --- | --- |
| RBBB Type(atypical,n%) | 55.0 | 83.3 | 0.003* |
| Transient RBBB,n(%) | 36.4 | 6.7 | 0.001* |
| STEMI,n(%) | 78.1 | 66.7 | 0.161 |
| QRS Duration(ms) | 158.22±23.59 | 198.83±10.72 | <0.001* |
| RV_1_  Interval(ms) | 115.23±12.20 | 128.83±8.579 | <0.001* |
| RV_6_-V_1_ Interval(ms) | 75.21±6.34 | 60.50±4.80 | <0.001* |
| QRS/RV6-V1 | 2.12±0.4 | 3.30±0.24 | <0.001* |

RBBB,right branch bundle block.STEMI,ST-segment elevation myocardial infarction.Statistically significant results were highlighted as *.

**
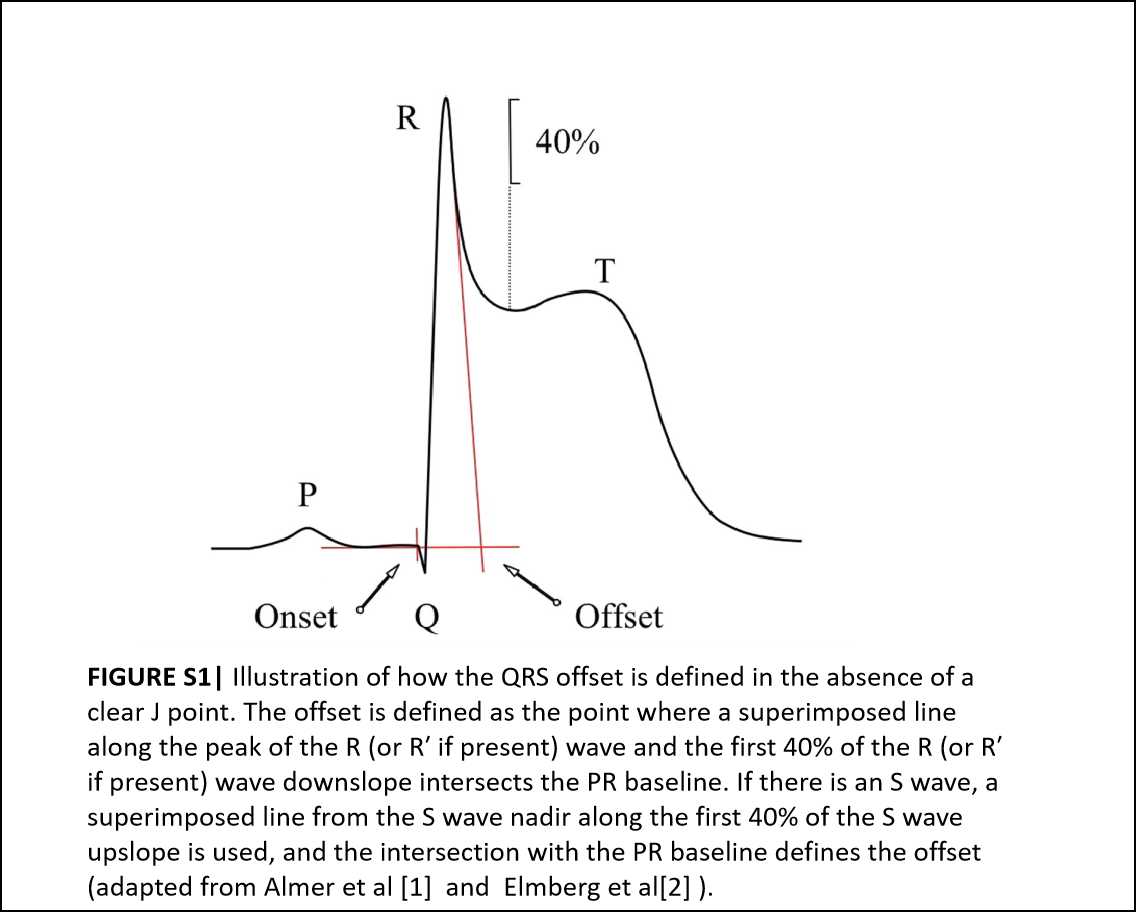
**

**1**

**
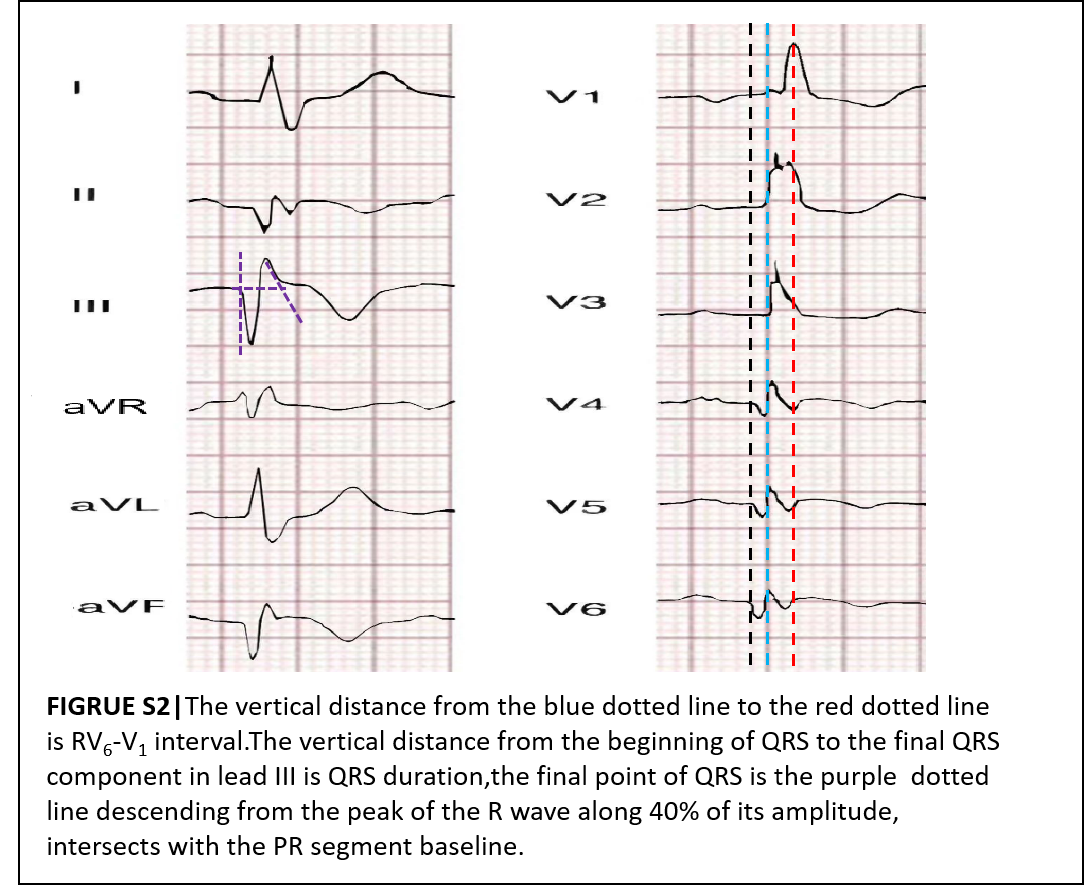
**


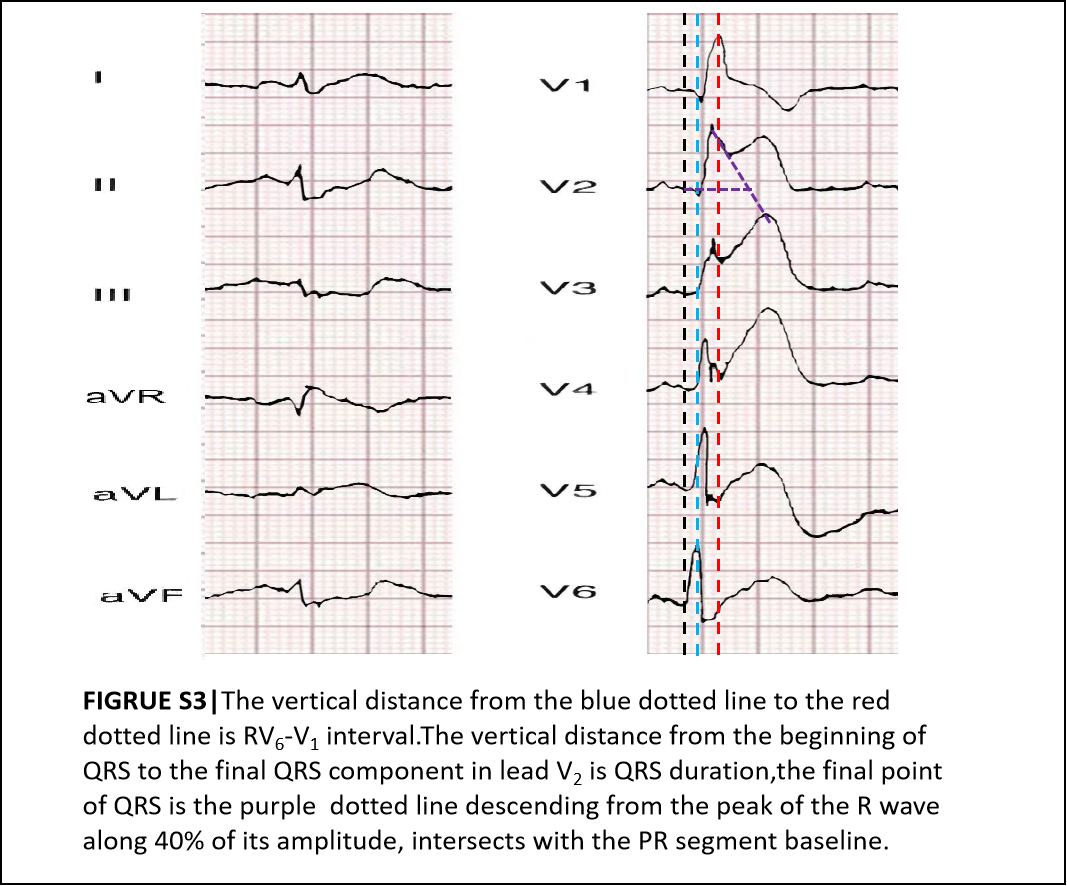


REFERENCE

1.Almer J,Jennings RB,Maan AC,Ringborn M,Maynard C,Pahlm O et al.Ischemic QRS prolongation as a biomarker of severe myocardial ischemia.J Electrocardiol.2016;49 (2)：139-47.doi:10.1016/j.jelectrocard.2015.12.010

2.Elmberg V,Almer J,Pahlm O,Wagner GS,Engblom H,Ringborn M.A 12-lead ECG-method for quantifying ischemia-induced QRS prolongation to estimate the severity of the acute myocardial event.J Electrocardiol.2016;49 (3)：272-7.doi:10.1016/j.jelectrocard.2016.02.001
